# Electrophysiological dataset from macaque visual cortical area MST in response to a novel motion stimulus

**DOI:** 10.1038/s41597-022-01239-z

**Published:** 2022-04-19

**Authors:** Benedict Wild, Amr Maamoun, Yifan Mayr, Ralf Brockhausen, Stefan Treue

**Affiliations:** 1 Cognitive Neuroscience Laboratory, German Primate Center - Leibniz Institute for Primate Research, Göttingen, Germany; 2 Göttingen Graduate Center for Neurosciences, Biophysics, and Molecular Biosciences (GGNB), University of Göttingen, Göttingen, Germany; 3 Faculty of Biology and Psychology, University of Göttingen, Göttingen, Germany; 4 Bernstein Center for Computational Neuroscience, Göttingen, Germany; 5 Leibniz ScienceCampus Primate Cognition, Göttingen, Germany

**Keywords:** Extrastriate cortex, Motion detection

## Abstract

Establishing the cortical neural representation of visual stimuli is a central challenge of systems neuroscience. Publicly available data would allow a broad range of scientific analyses and hypothesis testing, but are rare and largely focused on the early visual system. To address the shortage of open data from higher visual areas, we provide a comprehensive dataset from a neurophysiology study in macaque monkey visual cortex that includes a complete record of extracellular action potential recordings from the extrastriate medial superior temporal (MST) area, behavioral data, and detailed stimulus records. It includes spiking activity of 172 single neurons recorded in 139 sessions from 4 hemispheres of 3 rhesus macaque monkeys. The data was collected across 3 experiments, designed to characterize the response properties of MST neurons to complex motion stimuli. This data can be used to elucidate visual information processing at the level of single neurons in a high-level area of primate visual cortex. Providing open access to this dataset also promotes the 3R-principle of responsible animal research.

## Abstract (summary)

**Table:** 

Measurement(s)	spike train • eye movement measurement
Technology Type(s)	single-unit recording • eye tracking device
Factor Type(s)	direction, location, and speed of moving random dot patterns
Sample Characteristic - Organism	Macaca mulatta
Sample Characteristic - Environment	laboratory environment
Sample Characteristic - Location	Germany

## Background & Summary

Determining and quantifying the relation between physical stimuli and the neuronal responses they evoke is the most widely taken approach in sensory neuroscience, resulting in a variety of modeling and analysis approaches^1–3^ for determining the neural representation of stimulus parameters. Most studies focus on early, low-level visual areas, such as the retina^4–6^, lateral geniculate nucleus (LGN)^7–9^, or primary visual cortex (V1)^10–13^ of the mammalian brain. In primates, however, the visual system is hierarchically structured^14^ with neurons in “higher” areas – downstream of V1 – progressively showing more complex stimulus preferences^15^. For example, stimulus representations tend to become more “tolerant” or “invariant” for position, scale, and context from mid- to high-level areas in the ventral^16^ and dorsal^17^ pathways of the primate cortex. It has been proposed that conscious visual perception begins at the top of the processing hierarchy and that the quick, categorical recognition of objects (“forest before trees”) that guides most of our behavior relies predominantly on activity in the higher areas^18^. Therefore, a better understanding of the functional relationship between stimulus features and neuronal responses in mid- and high-level areas is essential for our understanding of sensation and brain function more broadly. Nevertheless, much fewer studies have focused on stimulus representations in mid-level or higher visual areas as compared to the rich literature on neural encoding of stimulus features in the early visual system (but see^16,19–21^ for examples). Even fewer such studies provide access to their data. For example, the Collaborative Research in Computational Neuroscience (CRCNS) website (crcns.org) offers 13 data sets with recordings from V1, but only 3 data sets of MT recordings^22–24^, one of V4 recordings^25^, and one of MST/VIP recordings^26^.

To address this shortage we provide a freely accessible and well curated comprehensive dataset from a neurophysiology study in macaque monkey visual cortex that includes a complete record of extracellular action potential recordings from the extrastriate medial superior temporal (MST) area, behavioral data, and detailed stimulus records. MST is a key area in the dorsal visual pathway that receives its major input from motion-sensitive area MT^27^ and is involved in the processing of complex motion as well as self-motion perception^17,21,28–30^. Notably, MST neurons have a number of complex features, such as position invariance^28^, and modeling work suggests that they perform a nonlinear integration of the output of MT neurons^21^. Models that include a nonlinearity are typically much more difficult to fit to data and require iterative procedures (as compared to, for example, simple linear-nonlinear (NL) cascade models for which the spike-triggered average corresponds to the maximum likelihood estimator of the neuron’s spatiotemporal receptive field^31^). We hope this dataset, which contains neuronal responses to different well-parameterized motion stimuli, can serve as a starting point to develop such models.

We provide the spiking activity of 172 MST neurons that were recorded across three different experiments. The datasets from the first two experiments (“Spatial Mapping” and “Tuning”) result from an elaborate and more systematic version of previous approaches (similar to, e.g.^28–30^) and can be used to determine the neurons’ spatial receptive fields as well as their tuning for direction and speed. For the “Spatial Mapping” experiment, small random dot patterns (RDPs) were sequentially presented in different locations across the screen, thus probing a neuron’s response to random motion across the visual field. The “Tuning” experiment presented RDPs that were moving at different speeds in different directions in linear motion and spiral motion space in multiple locations across each neuron’s spatial receptive field. As these two experiments take the standard approach for acquiring the stimulus-response function they can be considered the “ground truth” about visual response properties of MST neurons. The third experiment (“Reverse Correlation”) recorded spiking responses to a newly developed random dot motion stimulus which consists of a grid of positions, each with a direction and speed seed that determine the motion of dots in the vicinity of each grid location. Each seed has a Gaussian weighting field that determines its influence on the motion of surrounding dots. This creates an overall smooth, wave-like motion pattern. Each seed was assigned a new direction and speed every 100 ms, thus creating a temporal structure of the stimulus that makes it well-suited for spike-triggered analysis approaches^2^. We believe that providing this dataset to the scientific community will be of high value to theoretical, computational, and systems neuroscientists who are interested in visual processing beyond the retina, LGN, and V1.

## Methods

### Animal welfare statement

Research with nonhuman primates represents a small but indispensable component of neuroscience research^32,33^. The scientists in this study are aware and are committed to the responsibility they have in ensuring the best possible science with the least possible harm to the animals^34^. Providing the data collected in this study in a well-curated format and with open access to the scientific community contributes to this commitment. Such datasets ensure maximal transparency about study results and promote the accessibility of data, ensuring its best use. This is in line with the 3R-principle and international efforts to improve the reporting and accessibility of biomedical research data^35^.

All animal procedures of this study have been approved by the responsible regional government office (Niedersaechsisches Landesamt fuer Verbraucherschutz und Lebensmittelsicherheit, LAVES) under the permit numbers 3392 42502-04-13/1100 and 33.19-42502-04-18/2823. The animals were group-housed with other macaque monkeys in facilities of the German Primate Center in Goettingen, Germany in accordance with all applicable German and European regulations. The facility provides the animals with an enriched environment (including a multitude of toys and wooden structures^36,37^), natural as well as artificial light, exceeding the size requirements of the European regulations, and access to outdoor space. We have established a comprehensive set of measures to ensure that the severity of our experimental procedures falls into the category of mild to moderate, according to the severity categorization of Annex VIII of the European Union’s directive 2010/63/EU on the protection of animals used for scientific purposes^38^. Surgeries were performed aseptically under gas anesthesia using standard techniques, including appropriate perisurgical analgesia and monitoring to minimize potential suffering. The German Primate Center has several staff veterinarians who regularly monitor and examine the animals and consult on procedures. During the study, the animals had unrestricted access to food and fluid, except on the days where data were collected or the animal was trained on the behavioral paradigm. On these days, the animals were allowed unlimited access to fluid through their performance in the behavioral paradigm. Here the animals received fluid rewards for every correctly performed trial. Throughout the study, the animals’ psychological and veterinary welfare was monitored by the veterinarians, the animal facility staff and the lab’s scientists, all specialized in working with nonhuman primates. The three animals were healthy at the conclusion of our study and were subsequently used in other studies.

### Animals

Three male rhesus macaque monkeys (*Macaca mulatta*) contributed to this dataset. All animals had previously participated in other projects and were implanted with a custom-designed vertical titanium pin, orthopedically attached to the top of their skulls to allow to minimize head movements during the experiment, as well as with a recording chamber implanted over the parietal lobe based on a magnetic resonance imaging (MRI) scan. The recordings chambers were cylindrical with a diameter of 24 mm. Surgeries were conducted under general anesthesia and post-surgical care using standard techniques.

#### Monkey sun

**Fig. 1:**
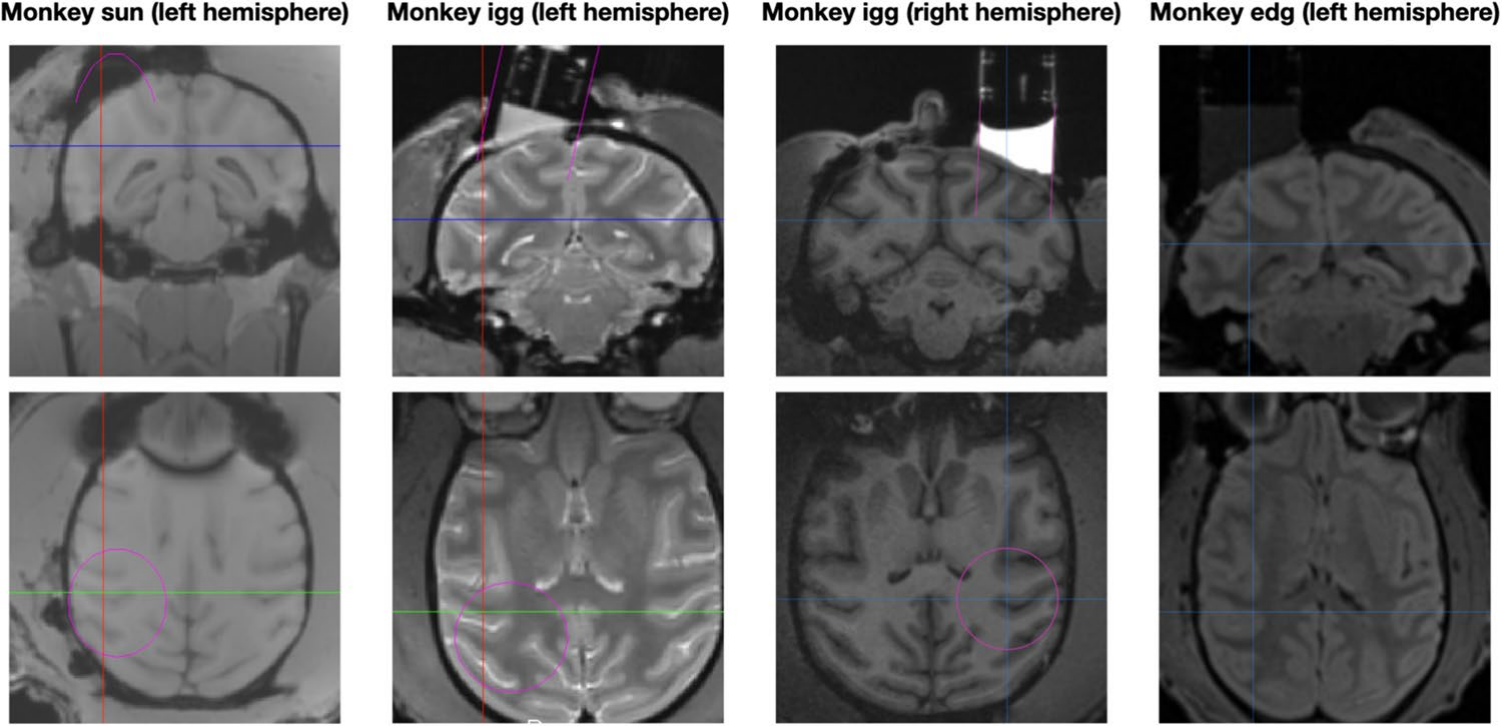
Implant locations of the recording chambers. The figure shows coronal (top row) and axial (bottom row) slices of MRI scans, displaying the location of the recording chamber in each of the 3 monkeys. Monkey igg was already implanted with a recording chamber over the left hemisphere when he joined the project (second column), but was re-implanted with another chamber over the right hemisphere (third column) during his participation in the project. The cross hairs indicate the location of MST. The pink circles in the first three pictures of the bottom row provide an estimate of the chamber position.

Monkey sun was 14–16 years old and weighed between 9.9 and 12.8 kg during the period of data collection. The monkey had previously participated in other projects and was already fitted with a titanium pin on the top of his skull and a chamber over the left hemisphere (stereotactic coordinates: mediolateral (ML): 15 mm left; anteroposterior (AP): 11 mm posterior, Fig. 1). The chamber was angled posterior with an inclination of 30° from the vertical. 82 of the 138 (59%) recordings included in this dataset come from Monkey sun.

#### Monkey igg

Monkey igg was 10–13 years old and weighed between 10.4 and 13.4 kg during the period of data collection. The monkey had previously participated in other projects and was already fitted with a titanium pin on the top of his skull and a chamber over the left hemisphere (coordinates: ML: 8 mm left; AP: 5 mm posterior, Fig. 1). The chamber was angled medial with an inclination of 18° from the vertical. During his participation in the project, the recording chamber over the left hemisphere was removed and a new vertically oriented chamber was implanted over the right hemisphere (coordinates: ML: 17 mm right; AP: 1 mm anterior, Fig. 1). 54 of the 138 (39%) recordings included in this dataset come from Monkey igg (26 from the left and 28 from the right hemisphere).

#### Monkey edg

Monkey edg was 14 years old and weighed between 8.0 and 9.2 kg during the period of data collection. The monkey had previously participated in other projects and was already fitted with a titanium pin on the top of his skull and a vertically oriented chamber over the left hemisphere (coordinates: ML: 13 mm left; AP: 0 mm, Fig. 1). 2 of the 138 dataset (2%) recordings included in this dataset come from Monkey edg.

### General experimental setup

Data were collected from two different experimental setups (setup A: Monkeys sun and edg as well as some data from Monkey igg; setup B: most data from Monkey igg). In both setups eye position was recorded binocularly with an Eyelink 1000 system (SR-Research, Ottawa, ON, Canada) at a sample rate of 500 Hz. The experiments were controlled by the open-source software MWorks (mworks-project.org) running on two Apple Macintosh computers (Apple Inc., Cupertino, CA, USA), a client and a server.

#### Setup A

During recordings a monkey was seated in a custom-built primate chair and viewed a 27” LCD monitor (XL2720T, BenQ, Taipei, Taiwan) from a distance of 57 cm in a dark room. The monitor had a resolution of 1920 × 1080 pixels, a refresh rate of 120 Hz, and covered 60° × 30° of the visual field. The luminance values of the background and the different stimuli are listed in Table 1.

**Table 1:** Luminance values of all stimuli in the two recording setups.

Stimulus Type	Setup A	Setup B
Background	17.3 cd/m^2^	1.0 cd/m^2^
White dots	82.7 cd/m^2^	31.3 cd/m^2^
Fixation point (bright)	13.4 cd/m^2^	1.9 cd/m^2^
Fixation point (dim)	8.9 cd/m^2^	0.6 cd/m^2^

#### Setup B

The conditions in this setup were the same as in setup A, except that a 171.5 × 107.2 cm back projection screen (dnp Black Bead, Karlslunde, Denmark) was viewed from a fixed distance of 102 cm. Stimuli were displayed via a projector (projection design, Fredrikstad, Norway) with a a resolution of 1920 × 1200 pixels and a refresh rate of 60 Hz.

### Task

Throughout all three experiments, monkeys performed a simple luminance change detection task. At the beginning of each trial a red fixation square (size: 0.2° × 0.21°) appeared. To make sure the different stimuli described below did not occlude the fixation square, a circular “mask” with a diameter of 1.5° of the same color as the background surrounded the fixation square. Monkeys could initiate the stimulus presentation by keeping their gaze within a square window of side length 3° around the fixation point (“fixation window”) and pressing a button attached to their primate chair. The luminance of the fixation point changed within a time window that was randomly selected from a uniform distribution ranging from 3 to 4.6 s after stimulus onset. The monkey had to respond to this luminance change within 600 ms (“reaction time window”) in order to receive a fluid reward (juice, tea, or water, depending on each monkey’s preferences). Monkeys sun and edg initiated a trial by briefly pressing the button and responded by pressing it again; Monkey igg kept the button pressed throughout the duration of the trial and responded by releasing it (these were the response patterns that the respective monkeys had been trained on in previous experiments). The exact luminance values before and after the change are listed in Table 1 for each setup. A trial could end in one of three ways: with a juice reward and a distinct sound if the monkey kept its gaze within the fixation window throughout the trial and responded to the luminance change within the reaction time window; with no reward and a different sound signaling an error if the monkey responded too early or too late (i.e., outside the reaction time window); or with no reward and a third sound if the monkey’s gaze moved outside the fixation window during the trial. The position of the fixation point was optimized in each recording session to ensure that as much of the receptive field as possible was covered by the display (within the constraints that fixation points too far out in the periphery made it difficult for the monkey to keep its gaze within the fixation window for a prolonged period of time).

### Experiments

On a given day the animal had to complete the experiments in the order “Spatial Mapping”, “Tuning”, “Reverse correlation”. The sum of all recordings on a given day are called one “recording session”. Given that the animal determined the number of trials performed each day, not all recording sessions contained complete recordings for all three experiments. Table 2 shows the number of recording sessions as well as the number of recorded neurons for each of the three experiments.

**Table 2:** Numbers of recording sessions and recorded units for each of the three experiments.

	Spatial Mapping	Tuning	Reverse Correlation
Number of sessions:	139	139	119
Number of recorded neurons:	172	172	150

#### Experiment 1 (Spatial mapping)

In Experiment 1, a small random dot pattern (RDP) was presented sequentially in different locations. The RDP consisted of white dots (cf. Table 1 for luminance values) with a radius of 0.2° that were randomly placed in a circular aperture with a radius of 1.5°. The dot density was 4 dots/deg^2^. Dots moved independently along linear trajectories in random directions and dots that moved out of the aperture were replotted at a random location within the aperture. Each presentation of the RDP for 50 ms constitutes one “sample”. For every sample, the location of the RDP was updated by assigning a random x-position in the range covering the entire width of the display (−28° to 28° in setup A, −30° to 30° in setup B) in steps of 2° and a random y-position in the range covering the entire height of the display (−16° to 16° in setup A, −20° to 20° in setup B) in steps of 2°, where the location [0°, 0°] is the center of the screen. The speed of the dots was randomly chosen from a range of 4°/s–24°/s for every sample. On trials that were not interrupted by fixation breaks or incorrect responses (see Task), more than 80 samples could be presented. In 76 cells, 10% of the sample presentations were blank (i.e., no stimulus other than the fixation point was shown on the screen for 50 ms). The purpose of these “blank presentations” was to calculate the baseline firing-rate of the cell in this task and to explore the cells’ responses to the random and quick location changes of the RDP on a longer timescale.

#### Experiment 2 (Tuning)

In Experiment 2, an RDP was presented sequentially in up to 5 different locations overlapping with the spatial receptive field as it had been determined by an online analysis of Experiment 1. The RDP consisted of white dots (cf. Table 1 for luminance values) with a radius of 0.2° that were randomly placed in a circular aperture with a radius ranging from 5° to 10°, depending on the size of the spatial receptive field. The dot density was 2 dots/deg^2^. The location, direction, and speed of the RDP were updated every 100 ms, the time period that constitutes one “sample” in Experiment 2. For most of the cells, the 5 locations were arranged in a cloverleaf, similar to a previous study^28^. In a minority of cells whose receptive field was on the edge or corner of the screen, the arrangement was different to ensure maximal coverage of the receptive field by the 5 RDP positions. For the position overlapping with the center of the receptive field, direction was chosen randomly from a set of 8 different translational and 8 spiral directions (see Table 3). For the other 4 locations, direction was randomly chosen from the 8 spiral directions. “Spiral space” is a one-dimensional space that includes radial, rotational, and spiral motion patterns. A direction in spiral space can be specified in degrees or radians, similar to translational directions. For an RDP that is moving in direction *α* in spiral space, the displacement of each dot is determined by updating its polar coordinates *r* (radius) and *θ* (angle) (with respect to the RDP’s center) as

$$r=r+s\ast {\rm{c}}{\rm{o}}{\rm{s}}(\alpha )$$

$$\theta =\theta +s\ast {\rm{s}}{\rm{i}}{\rm{n}}(\alpha )$$

where *s* is a constant that depends on speed. Thus, for *α* = 0°, the distance of each dot from the RDP’s center (i.e., it’s radius, *r*) increases maximally (because cos(0) = 1) and the angle does not change at all (because sin(0) = 0), which results in expansion (all dots moving away from the center). For *α* = 90°, each dot’s radius remains the same (because cos(90) = 0) and the angle increases maximally (because sin(90) = 1), which results in clockwise rotation. Table 3 shows an overview of the 8 directions in translational and spiral space. The speed of each RDP was randomly selected for every sample from one of six discrete values evenly spaced from 4°/s–24°/s. During spiral motion, RDPs had a speed gradient with the specified speed determining the speed of dots at a distance of 1° from the RDP’s center. As in Experiment 1, 10% of samples were “blank samples” (i.e., no RDP was shown) in 76 cells.

**Table 3:** Definitions of translational and spiral motion directions.

Direction in degrees	Linear Motion	Spiral Motion
0°	Up	Expansion
45°	Diagonal up and right	Clockwise outward spiral
90°	Right	Clockwise rotation
135°	Diagonal down and right	Clockwise inward spiral
180°	Down	Contraction
225°	Diagonal down and left	Counterclockwise inward spiral
270°	Left	Counterclockwise rotation
315°	Diagonal up and left	Counterclockwise outward spiral

Note that the definition for translational motion deviates from the geometric convention where 0° is rightward and angle increases in counterclockwise direction; instead 0° is defined as upward motion and angle increases in the clockwise direction.

#### Experiment 3 (Reverse correlation)

In Experiment 3, a newly developed large, rectangular RDP was presented, which we call RC stimulus (“reverse correlation stimulus”). What the monkey sees is a smooth, wave-like pattern of moving dots (a video of an example trial is provided in the repository, see below for detailed information). Technically, the stimulus consists of 10 × 15 square segments, each with a side length of 3°, resulting in an overall size of 30° × 45°. Each segment is assigned a random translational direction and a random speed from 0°/s–20°/s every 100 ms, the time period that constitutes one “sample” in Experiment 3. The center of each segment provides a Gaussian weighting field which determines its influence on the dots. Each dot’s direction and speed are calculated as

$$f(x,y)=\frac{1}{n}\frac{{\sum }_{i}^{n}{p}_{i}{\rm{e}}{\rm{x}}{\rm{p}}\left(-\left(\frac{{(x-{x}_{i})}^{2}}{2{\sigma }_{1}^{2}}+\frac{{(y-{y}_{i})}^{2}}{2{\sigma }_{2}^{2}}\right)\right)}{{\sum }_{i}^{n}{\rm{e}}{\rm{x}}{\rm{p}}\left(-\left(\frac{{(x-{x}_{i})}^{2}}{2{\sigma }_{1}^{2}}+\frac{{(y-{y}_{i})}^{2}}{2{\sigma }_{2}^{2}}\right)\right)}$$

with the following variables:

*n:* number of neighboring segments ranging from 3 to 9

*p*_*i*_: parameter (direction or speed) in question of segment

*x*, *y:* coordinates of the dot in degrees relative to the fixation point

*x*_*i*_, *y*_*i*_: coordinates of center of segment *i* relative to the fixation point

*σ*_1_: standard deviation of the Gaussian filter along its first axis

*σ*_2_: standard deviation of the Gaussian filter along its second axis

The standard deviations of the Gaussian filter *σ*_1_ and *σ*_2_ were both set to a value of 1.2° in all experiments. Each segment contained 10 dots with a radius of 0.2° that were randomly placed inside the segment (cf. Table 1 for luminance values), resulting in a dot density of 1.1 dots/deg^2^ and a total number of 1500 dots for the entire stimulus. For 47 recording sessions conducted with Monkey igg, parts of the RC stimulus were masked with rectangles of the same color and luminance as the background, so as to effectively reduce the size of the stimulus to 6 × 9 square segments (overall stimulus size of 18° × 27°).

### Neural recording setup

For each recording session, between one and three microelectrodes (Thomas Recording, Giessen, Germany) were advanced into the bank of the superior temporal sulcus, targeting area MST using microdrives. The microdrive was mounted at the beginning of each session onto the recording chamber and a x-position (on the medio-lateral axis) and a y-position (on the anterior-posterior axis) of the single or central electrode were determined. For most of the recording sessions, electrode depth position was controlled with a 3 electrode or a 5 electrode “Mini Matrix” system (Thomas Recording, Giessen, Germany), where electrodes are advanced using a rubber tube mechanical system. For a small subset of recordings from the left hemisphere of Monkey igg (a total of 22 recordings), we used the Model 650 single electrode Micropositioner (David Kopf Instruments, Tujunga, CA, USA), where the electrode is advanced using a hydraulic system. Signals from the electrodes were amplified and recorded with a sampling rate of 40 kHz and 16-bit precision using an Omniplex acquisition system (Plexon, Dallas, TX, USA).

### Data preprocessing

Action potentials (“spikes”) were identified using OfflineSorter V4 (Plexon, Dallas, TX, USA). The raw data was filtered with a 6-pole Bessel high-pass at a cut-off frequency of 250 Hz and spike waveforms were detected based on a manually determined threshold. These waveforms were then manually split into clusters based on different features as implemented in the software, including the first three principal components of the waveforms, the maximum and minimum voltage amplitude across the entire waveform length (“peak” and “valley”), or the waveform energy. For each recording, features were chosen according to the best separation between clusters. Note that for the large majority of recordings, there was only one unit recorded so that this procedure predominantly served the purpose to separate the signal from background noise. Only in 29 sessions 2 units and in 2 sessions 3 units were recorded simultaneously, which required actual sorting of waveforms as belonging to different units. Behavioral and stimulus parameters of each recording session were originally stored in the MWK format as a stream of MWorks events (mworks-project.org). The lists contain the time, the name of the event, and data associated with the event. To increase data accessibility, the relevant parameters of each experiment, together with online and offline sorted spikes, were converted into the HDF5 format (see Usage Notes).

## Data Records

All data and supplementary material are publicly available via the German Neuroinformatics Node (G-Node, http://www.g-node.org). We provide a complete set in a permanently archived format (~27 GB), and in addition maintain a repository for possible future updates of the data and the supplementary material^39^. The datasets are provided in the Hierarchical Data Format 5 (HDF5). We chose the HDF5 format because it is a portable and self-describing file format where data and metadata can be passed along in one file, in accordance with the FAIR guiding principles for scientific data management^40^. The file structure of HDF5 files includes only two major types of objects - datasets and groups. Datasets are homogeneous *n*-dimensional arrays, and groups are container structures which hold datasets and/or groups. Metadata can be added to datasets and groups as attributes. The HDF5 data model, file format, API, library, and tools are open and distributed without charge (https://www.hdfgroup.org/solutions/hdf5) and the content of HDF5 files can be directly viewed in the freely available HDF Viewer (https://www.hdfgroup.org/downloads/hdfview/). Furthermore, the format can easily be converted and is therefore accessible using multiple widely used programming languages, such as MATLAB, Python, R, and Julia. Because of these advantages this format has seen increased popularity in recent years for the storage of neuroscience data^41–45^.

Data from each of the three experiments are organized in three folders: *MSTm* contains data from Experiment 1 (“Spatial Mapping”), *MSTt* contains data from Experiment 2 (“Tuning”), and *MSTn* contains data from Experiment 3 (“Reverse Correlation”). For each recording session there are two files: a’task.h5’ file contains the stimulus parameters, online and offline sorted spikes, and trial descriptions; an’eye.h5’ file stores gaze position and pupil size. For some recording sessions, no data is available for Experiment 3 because of technical issues during the recording, because the recorded neuron was lost partway through the recording session, or because the monkey would not do enough trials on that day. Metadata about each recording session is provided as a separate tab separated value (tsv) file. Information is provided in a table, where each row describes one recording session and the columns are explained in Table 4. An additional table (data_description) provides definitions of all the variables. Lastly, the repository contains three videos in the.mp4 format of example trials from each of the three experiments (Exp1_spatial_mapping.mp4, Exp2_tuning.mp4, and Exp3_reverse_correlation.mp4).

**Table 4:** Description of the meta data as provided in a separate tsv file.

Column heading	Description
format	Format version of the meta data table (“1.0” in call cases in this project)
recording_session	A running index of recording sessions
experimenter	The experimenter who recorded the data (“amm” for A.M. or “bew” for B.W.)
date	The date of the recording
monkey	The monkey (“sun”, “igg”, or “edg”)
hemisphere	The hemisphere from which the data was recorded (“left” or “right”)
chamber	A numerical identifier of the recording chamber on a hemisphere (“1” in all cases in this project)
session_number	A running counter of recording sessions for a given monkey (recorded as strings to include leading zeros which are needed to specify filenames)
daily_count	A running counter of separate recording attempts on a given day
hardware	The recording setup in which the file was recorded (“A” or “B”) and the micropositioner that was used for the recording session (either “MM1” for the 5 electrode mini matrix, “MM2” for the 3 electrode mini matrix or “Kopf” for the hydraulic Model 650 Micropositioner, see Neural recording setup)
offline_units_n	*n* (here: *n* = 3) columns with an identifier for up to *n* offline-sorted single cells. The digits before the decimal point specify the electrode while digits after the decimal point specify different offline sorted units recorded on the same electrode. For example, 34.1 and 34.2 are two units recorded on the same electrode that were sorted offline, whereas 34.1 and 35.1 are two units that were recorded on separate electrodes
ML_n	*n* (here: *n* = 3) columns with the stereotaxic coordinates on the mediolateral axis of the electrode for up to *n* offline-sorted single cells that were identified in the columns “offline_units_n” (in mm)
AP_n	*n* (here: *n* = 3) columns with the stereotaxic coordinates on the anteroposterior axis of the electrode for up to *n* offline-sorted single cells that were identified in the columns “offline_units_n” (in mm)
depth_n	*n* columns (here: *n* = 3) with depth positions of the electrode for up to *n* offline-sorted single cells that were identified in the columns “offline_units_n”, where 0 is the surface of the dura mater (in *μ*m)
Exp_MSTm_tt, Exp_MSTt_tt, Exp_MSTn_tt	One column for each experiment (MSTm, MSTt, MSTn) that specifies the total number trials in this file (‘tt’ = ‘total trials’)
Exp_MSTm_st_n, Exp_MSTt_st_n, Exp_MSTn_st_n	*n* columns (here: *n* = 3), for each combination of experiment (MSTm, MSTt, MSTn) and up to *n* units that specify the range of trials on which spikes were recorded (‘st’ = ‘spike trials’)
notes	Additional information about a recording session that is not recorded in any of the previous columns.

## Technical Validation

We performed a number of plausibility checks to ensure the quality of the data and to rule out some potential problems that could bias the analysis of the data. First, we verified that the following is true:

- There are no negative spike times.
- Trial end times are always later than trial start times, i.e., all trials have a positive duration.
- The number of timestamps for every event is identical to the number of event values (see Usage Notes for a detailed description of event values and timestamps).

### Trial duration

Individual trials can be arbitrarily short, because a trial was aborted immediately if the monkey’s gaze exited the fixation window or the monkey made a premature response (see Methods). Trials can also be arbitrarily long, when the monkey pressed a button in response to the luminance change of the fixation point (see Methods) and then kept it pressed, rather than releasing it quickly. We therefore checked the time from trial start until the assignment of a trial outcome (hit, failure, or fixation break) in “hit”-trials, as these were not interrupted by fixation breaks or premature responses. The durations of these trials ranged from 3112 ms to 4604 ms (mean ± SD: 3832 ± 312 ms), which is in accordance with the values that had been specified (3.0 to 4.6 s).

### Firing rate

In each of the three experiments, trials consist of rapidly changing presentations of randomly selected stimuli. On any given trial this includes stimuli that drive the cell’s spiking activity strongly as well as stimuli that drive it hardly at all, so that the firing rate within a trial is expected to remain stable on average, without any systematic deviation over time. We do expect the firing rate per trial to be higher in the “Tuning” experiment than in the “Spatial Mapping” experiment, because all stimuli were presented inside the receptive field and the stimuli were typically larger and contained coherent motion (compared to the small, incoherent motion RDPs used for “Spatial Mapping”, see Methods). And indeed, a paired *t*-test showed a highly significant difference in the average firing rate of “Spatial Mapping” trials (15.64 spikes/s) and “Tuning” trials (19.80 spikes/s) (*t*(138) = 5.98, *p* < 0.001). Across all recordings and all experiments, 1160 trials that last at least 500 ms (0.78%) have 0 spikes. Across all units, all experiments, and all trials that last at least 500 ms, the average firing rate (calculated per trial) is 16.80 spikes/s (SD: 12.45 spikes/s) and the highest firing rate calculated for one individual trial is 162 spikes/s. This value seems quite high and came from a trial that was quite short (833 ms) but contained a very large number of spikes (135). As we note in the next paragraph, some of these outliers might be caused by incorrectly classified waveforms during spike sorting, but across the entire data set, they are very rare.

### Interspike intervals

The longest interspike interval (ISI) across all files is 15 s, but only 37 ISIs are longer than 4 s. which we consider realistic and plausible. Across all recordings and experiments, there are 1413 ISIs (0.02%) that are longer than 2 s and 8319 interspike intervals (0.13%) that are shorter than 1 ms. Because of small shifts in the position of the electrode over time, the quality of the voltage signal can change drastically. If the waveform changes too much, spike sorting might miss some spikes, misclassify them as belonging to a different unit, or incorrectly classify waveforms as belonging to the unit event though they do not. While it is nearly impossible to rule out misclassifications for sure, we believe that the information we provide here about firing rates and interspike intervals strongly suggests that this issue is negligible in our dataset. However, it should be kept in mind that each individual spike has a certain, albeit very low, probability of being misclassified which might be relevant for certain types of analyses.

In summary, we believe that these tests provide ample evidence for the quality of the data.

## Usage Notes

Our dataset is organized by experiment. Within each experiment folder there are two files for every recording session. Files ending in’-task.h5’ contain information about the stimuli, behavior, and electrophysiological recordings; files ending in’-eye.h5’ contain eye tracking data. Each of these file pairs contains the complete data from all the trials the monkey completed. Here, we provide a general overview of this data structure and how variables of interest can be accessed. More specific examples for how this can be achieved in MATLAB are given by the example code (see Code availability). In both files the data is structured as a series of “events”. There are five types of events that can be recognized by their names (the first four types only occur in -task.h5 files and the last type only occurs in -eye.h5 files):

- events that describe stimulus features (e.g., the direction or speed of a random dot pattern) start with STIM_There are 10 different stimuli with distinct prefixes:
  - STIM_background describes features of the background (all three experiments)
  - STIM_fixationPoint describes features of the fixation square (all three experiments)
  - STIM_MappingProbe describes features of the RDP in the Spatial Mapping experiment (Experiment 1 only)
  - STIM_mask describes features of the circular mask around the fixation square (all three experiments)
  - STIM_nDimRDP describes features of the RC stimulus (Experiment 3 only)
  - STIM_RecmaskBottom, STIM_RecmaskLeft, STIM_RecmaskRight, and STIM_RecmaskTop desribe features of the four rectangular masks that covered part of the RC stimulus in some of the recordings as described in the section Experiment 3 (Reverse Correlation) (Experiment 3 only)
  - STIM_TuningProbe describes features of the RDP in the Tuning experiment (Experiment 2 only)
- events that describe input/output variables (e.g., button press or reward delivery) start with IO_
- events that describe trial parameters (such as trial start or trial type) start with TRIAL_
- events that relate to action potential recordings start with SPIKE_
- events that describe eye tracking data start with EYE_

More detailed definitions of all events can be found in the file data_description in the repository. Each event has a value and a timestamp, specifying the time in microseconds since the MWorks server was started on the day of the recording session. Each task-file (i.e., files ending in ‘_task.h5’) includes two structures – event_value, and event_time – of equal length that contain a field of values or timestamps for every event. As a concrete example, in the recording session “amm-MSTm-sun-120-01 + 01” the first value that is assigned to the event TRIAL_start is the integer 1 at 59453797 *μ*s (about 1 minute) after the MWorks server had been started. This information is saved in amm-MSTm-sun-120-01 + 01-task.h5 as event_value/TRIAL_start[0] = 1 and event_time/TRIAL_start[0] = 59453797 (note that if reading the data into Matlab, the index needs to be 1, rather than 0). In other words, event_time/TRIAL_start is an array whose length equals the number of trials and whose entries specify the start time of each trial. Correspondingly, event_value/TRIAL_start is an array of the values of TRIAL_start at each timestamp. In the case of TRIAL_start, these values are the trial numbers. For events that describe stimulus attributes, such as the event STIM_MappingProbe_posX – which describes the x-coordinate of the random dot pattern that was presented in Experiment 1 (see Methods) – this would be a list of x-positions. A description of events is included in each file in the form of attributes and additionally provided as a separate table in the repository for easy reference. The ‘eye’-files are structured in the same way, with event_value/EYE_x_dva, for example, specifying the x-coordinate of the monkey’s gaze and event_time/EYE_x_dva specifying the corresponding time stamps, which are synchronized with the time stamps of the corresponding task-file.

## Data Availability

We provide MATLAB code that serves two purposes: (a) to give examples of how the data can be accessed; and (b) to perform some elementary data validation analyses. The code is available along with the data in the repository. We briefly summarize the MATLAB scripts that we provide along with the data. ***h5_extract.*** h5_extract() is a function that takes as its input the name of an HDF5 file, a cell array of strings that specify the parameters of the experiment that are to be extracted (for this dataset, this should always be event_value, and event_time), and a cell array of strings that specify the events that are to be extracted (typically the list of event names that can be extracted from the HDF5 itself, as demonstrated in the example scripts). The output of the function are two MATLAB structure arrays - event_value and event_time - which correspond to the groups in the HDF5 file of the same name. Each field name of the structure arrays is the name of an event and the content in the field corresponds to the values and times associated with the event, respectively. Once extracted, the data are ready for visualization and analysis. The scripts technical_validation, example_raster_fr, and example_trial_histogram demonstrate the use of the h5_extract() function to read in all events saved in an HDF5 file. ***technical_validation.*** The technical_validation script was used to perform all the plausibility checks described in the section “Technical Validation”. The script prints all the statements from that section of the manuscript that contain quantitative information about the data (such as, for example, average firing rate) to the console. ***example_raster_fr.*** To demonstrate how individual spike times can be accessed and visualized, the script creates a raster plot of spike trains and a scatter plot of firing rates per trial (for trials lasting at least 500 ms), colored by trial outcome, for one example file. The example file is specified in the first line of the script and can easily be changed by the user. The raster plot also includes the time of reward delivery and the end of each trial. ***example_trial_histogram.*** To demonstrate how multiple files can be accessed for population analyses, a histogram of the number of trials across recording sessions is produced for each of the three experiments and color-coded by monkey. ***example_probe_time.*** This script contains a function, probe_time(), which extracts the values of all events at a given time point, as well as some additional code that illustrates the use of the function. ***example_rc_stim_extraction.*** This script demonstrates how the direction and speed values of the RC stimulus can be extracted and in particular, how those segments that were masked in some of the files (see Methods) can be determined. ***example_eye_data.*** To demonstrate how the eye tracking data can be accessed, this function plots the x- and y-position of the monkey’s gaze as well as the size of the right and left pupil for the first *n* timesteps (where *n* can be specified by the user, default is 100). ***example_spatial_mapping_analysis and example_tuning_analysis.*** To demonstrate how spiking activity can be related to stimulus features, these two functions plot firing rate as a function of probe location in the Spatial Mapping experiment (example_spatial_mapping_analysis) or as a function of motion direction and speed in the Tuning experiment (example_tuning_analysis).

## References

[1] Paninski, L., Pillow, J. & Lewi, J. Statistical models for neural encoding, decoding, and optimal stimulus design. In Cisek, P., Drew, T. & Kalaska, J. F. (eds.) *Progress in Brain Research*, **165**, 493–507, 10.1016/S0079-6123(06)65031-0 (2007). 10.1016/S0079-6123(06)65031-0 17925266

[2] Schwartz O, Pillow JW, Rust NC, Simoncelli EP (2006). Spike-triggered neural characterization. Journal of Vision.

[3] Wu MC-K, David SV, Gallant JL (2006). Complete Functional Characterization of Sensory Neurons by System Identification. Annual Review of Neuroscience.

[4] Liu JK (2017). Inference of neuronal functional circuitry with spike-triggered non-negative matrix factorization. Nature Communications.

[5] Maheswaranathan N, Kastner DB, Baccus SA, Ganguli S (2018). Inferring hidden structure in multilayered neural circuits. PLOS Computational Biology.

[6] Pillow JW (2008). Spatio-temporal correlations and visual signalling in a complete neuronal population. Nature.

[7] Cai D, Deangelis GC, Freeman RD (1997). Spatiotemporal Receptive Field Organization in the Lateral Geniculate Nucleus of Cats and Kittens. Journal of Neurophysiology.

[8] Dan Y, Alonso J-M, Usrey WM, Reid RC (1998). Coding of visual information by precisely correlated spikes in the lateral geniculate nucleus. Nature Neuroscience.

[9] Solomon SG, Tailby C, Cheong SK, Camp AJ (2010). Linear and Nonlinear Contributions to the Visual Sensitivity of Neurons in Primate Lateral Geniculate Nucleus. Journal of Neurophysiology.

[10] Jones JP, Palmer LA (1987). The two-dimensional spatial structure of simple receptive fields in cat striate cortex. Journal of Neurophysiology.

[11] Park M, Pillow JW (2011). Receptive Field Inference with Localized Priors. PLoS Computational Biology.

[12] Rust NC, Schwartz O, Movshon JA, Simoncelli EP (2005). Spatiotemporal Elements of Macaque V1 Receptive Fields. Neuron.

[13] Touryan J, Lau B, Dan Y (2002). Isolation of Relevant Visual Features from Random Stimuli for Cortical Complex Cells. Journal of Neuroscience.

[14] Felleman DJ, Van Essen DC (1991). Distributed hierarchical processing in the primate. cerebral cortex. Cerebral Cortex.

[15] Treue S (2003). Climbing the cortical ladder from sensation to perception. Trends in Cognitive Sciences.

[16] Rust NC, DiCarlo JJ (2010). Selectivity and Tolerance (“Invariance”) Both Increase as Visual Information Propagates from Cortical Area V4 to IT. The Journal of Neuroscience.

[17] Wild B, Treue S (2021). Primate Extrastriate Cortical Area MST: A Gateway between Sensation and Cognition. Journal of Neurophysiology.

[18] Hochstein S, Ahissar M (2002). View from the top: Hierarchies and reverse hierarchies in the visual system. Neuron.

[19] Rust NC, Mante V, Simoncelli EP, Movshon JA (2006). How MT cells analyze the motion of visual patterns. Nature Neuroscience.

[20] Yamane Y, Carlson ET, Bowman KC, Wang Z, Connor CE (2008). A neural code for three-dimensional object shape in macaque inferotemporal cortex. Nature Neuroscience.

[21] Mineault PJ, Khawaja FA, Butts DA, Pack CC (2012). Hierarchical processing of complex motion along the primate dorsal visual pathway. Proceedings of the National Academy of Sciences of the United States of America.

[22] Cui Y, Liu LD, Khawaja FA, Pack CC, Butts DA (2013). CRCNS.org.

[23] Nishimoto S, Gallant JL (2018). CRCNS.org.

[24] Niknam K (2018). CRCNS.org.

[25] Smith M (2020). Figshare.

[26] Gu Y (2018). CRCNS.org.

[27] Born RT, Bradley DC (2005). Structure and Function of Visual Area MT. Annual Review of Neuroscience.

[28] Graziano MSA, Andersen RA, Snowden RJ (1994). Tuning of MST neurons to spiral motions. Journal of Neuroscience.

[29] Saito H-A (1986). Integration of direction signals of image motion in the superior temporal sulcus of the macaque monkey. Journal of Neuroscience.

[30] Tanaka K (1986). Analysis of local and wide-field movements in the superior temporal visual areas of the macaque monkey. Journal of Neuroscience.

[31] Pillow, J. W. Likelihood-Based Approaches to Modeling the Neural Code. In Doya, K., Ishii, S., Pouget, A. & Rao, R. P. N. (eds.) *Bayesian Brain: Probabilistic Approaches to Neural Coding*, 53–70, 10.7551/mitpress/9780262042383.003.0003 (MIT Press, 2007).

[32] Buffalo EA, Movshon JA, Wurtz RH (2019). From basic brain research to treating human brain disorders. Proceedings of the National Academy of Sciences of the United States of America.

[33] Treue, S. & Lemon, R. The indispensable contribution of nonhuman primates to biomedical research. In Robinson, L. & Weiss, A. (eds.) *Nonhuman Primate Welfare*, 10.1007/978-3-030-82708-3 (Springer, Cham, 2022).

[34] Roelfsema PR, Treue S (2014). Basic Neuroscience Research with Nonhuman Primates: A Small but Indispensable Component of Biomedical Research. Neuron.

[35] Percie du Sert N (2020). The ARRIVE guidelines 2.0: Updated guidelines for reporting animal research. PLOS Biology.

[36] Berger M (2018). Standardized automated training of rhesus monkeys for neuroscience research in their housing environment. Journal of Neurophysiology.

[37] Calapai A (2016). A cage-based training, cognitive testing and enrichment system optimized for rhesus macaques in neuroscience research. Behavior Research Methods.

[38] Pfefferle D, Plümer S, Burchardt L, Treue S, Gail A (2018). Assessment of stress responses in rhesus macaques (Macaca mulatta) to daily routine procedures in system neuroscience based on salivary cortisol concentrations. PLos One.

[39] Wild B, Maamoun A, Mayr Y, Brockhausen R, Treue S (2021). G-Node.

[40] Wilkinson MD (2016). The FAIR Guiding Principles for scientific data management and stewardship. Scientific Data.

[41] Brochier T (2018). Massively parallel recordings in macaque motor cortex during an instructed delayed reach-to-grasp task. Scientific Data.

[42] Diggelmann R, Fiscella M, Hierlemann A, Franke F (2018). Automatic spike sorting for high-density microelectrode arrays. Journal of Neurophysiology.

[43] Herz AV, Meier R, Nawrot MP, Schiegel W, Zito T (2008). G-Node: An integrated tool-sharing platform to support cellular and systems neurophysiology in the age of global neuroinformatics. Neural Networks.

[44] Zehl, L. *et al*. Handling Metadata in a Neurophysiology Laboratory. *Frontiers in Neuroinformatics* **10**, 10.3389/fninf.2016.00026 (2016). 10.3389/fninf.2016.00026 PMC4949266 27486397

[45] Stoewer, A., Kellner, C., Benda, J., Wachtler, T. & Grewe, J. File format and library for neuroscience data and metadata. *In Neuroinformatics 2014*, 10.3389/conf.fninf.2014.18.00027 (INCF, Leiden, Netherlands, 2014).

